# Crosslinked Decellularized Porcine Pericardium as a Substrate for Conjunctival Reconstruction

**DOI:** 10.1155/2022/7571146

**Published:** 2022-03-15

**Authors:** Fangyuan Chen, Jingyue Deng, Lishi Luo, Ying Zhu, Yuying Dong, Yuanting Yang, Rijia Zhang, Jian Chen, Qing Zhou

**Affiliations:** ^1^Department of Ophthalmology, The First Affiliated Hospital of Jinan University, Guangzhou 510630, China; ^2^The Fifth Affiliated Hospital of Southern Medical University, Guangzhou 510900, China; ^3^Shenzhen Eye Hospital, Affiliated Hospital of Jinan University, Shenzhen 518040, China

## Abstract

Seeking for suitable conjunctival reconstruction substitutes to overcome the limitations of current substitutes, such as amniotic membrane, is urgent. Decellularized tissues have become a promising strategy for tissue engineering. In this study, we prepared decellularized porcine pericardium (DPP) scaffolds by the phospholipase A2 method and crosslinked them with aspartic acid (Asp) and human endothelial growth factor (hEGF) to enhance biological performance on the DPP, obtaining DPP-Asp-hEGF scaffolds. In vitro DPP showed lower apoptosis, highly desirable, well preservation of extracellular matrix components, and favorable macro-microstructure, which was confirmed by histology, immunofluorescence, electron microscopy, collagen and DNA quantification, and cytotoxicity assay, compared to the native porcine pericardium (NPP). The crosslinked efficacy of the DPP-Asp-hEGF was furtherer verified by in vitro experiments with Fourier transform infrared (FTIR) and X-ray diffraction (XRD). Through animal models of conjunctiva defect model, the DPP-Asp-hEGF revealed a closed, multilayer epithelium with an equal amount of goblet cells and no indication for conjunctival scarring after 28 days, compared to amniotic membrane (AM) groups and sham groups. These results suggested that DPP-Asp-hEGF can offer a good conjunctival reconstructive substitute both in structure and in function.

## 1. Introduction

The conjunctiva is essential in the healthy ocular surface and provides a physical and an immunological barrier promoting innate and adaptive immunity [1]. Severe conjunctival damage, such as ocular cicatricial pemphigoid and Stevens-Johnson syndrome, trauma, and thermal or chemical burns, leads to severe ocular surface scarring and need a suitable substitute for surgical reconstruction of functional fornixes and conjunctiva. Treatments that rely on the autologous conjunctiva are difficult, especially in cases with large conjunctival defects or severe inflammatory surface disorders in both eyes. Furthermore, investigators are also increasingly aware of the limitations of autologous tissue substitutes, including the risk of virus transmission and the absence of goblet cells of donors [2]. For these reasons, the development of a suitable alternative graft material quality for conjunctiva has become the focus of increasing attention.

Collagen-based extracellular matrix derived from the human placenta, the amniotic membrane (AM), has great advantages in readily available, inexpensive, naturally biocompatible [3], and promotion of epithelialization [4] and has frequently been used in the conjunctival repair of the ocular surface over the past decade. However, in cases of severe ocular surface inflammation, AM can degrade quickly leading to decreased epithelialization and prone to scarring and cauterization recurrently by cytokines and growth factors [5]. Additionally, AM transplantation requires costly screenings and disease transmission cannot be completely excluded [4]. To address these limitations, human decellularized amniotic membrane (dAM) has been explored and showed great potential in supporting tissue reconstruction (e.g., wound dressing [6] and limbal stem cell deficiency [7]). However, the weak surgical usability has the limited surgical operation and suturing, and rapid degradation leading to reoperation limited its widespread use in the clinic [7].

Among several biomaterials, extracellular matrix (ECM), derived from donor tissue, has attractive superiority in tissue engineering and regenerative medicine, compared to synthetic substrates. As a promising biological scaffold, ECM provides not only the native 3D structure for cellular growth but also bioactive components from naive tissue for cellular migration, proliferation, morphogenesis, adhesion, and differentiation [8]. Decellularized extracellular matrix (ECM) scaffolds, including decellularized human corneal stroma [9], decellularized porcine cornea (DPC) [10, 11], decellularized porcine conjunctiva [12, 13] and decellularized human conjunctiva [13], decellularized amniotic membrane [14], and decellularized bovine pericardium [15], have been tested in previous studies for ocular reconstruction.

Although the decellularization technique has been studied for a long time, there is no standard protocol. It is suggested to make a different protocol of decellularization for each specific tissue and it is necessary to complement with a postdecellularization process, to reduce the loss and damage of ECM in the process of decellularization [16]. Previous studies showed that crosslinking can improve mechanical, structural, and biological properties and biodegradation rate [16, 17]. 1-Ethyl-3-(3-dimethyl aminopropyl) carbodiimide (EDC) is a low-toxicity or not cytotoxic chemical that has been widely applied for crosslinking [16, 18]. EDC, a carboxylic acid group activation, could form amide bonds by coupling with reactive intermediates and the amino group [19]. N-Hydroxysuccinimide (NHS) can enhance the stability of EDC crosslinked products by reacting to generate more stable esters; thus, crosslinking with EDC/NHS can result in a highly aligned fibrillar structure with banding similar to native collagen [20]. Some reports showed aspartic acid (Asp) crosslinked with epidermal growth factor (EGF) on the scaffold by activating carboxylic acid groups in collagen [21, 22].

In this study, we use EDC/NHS agent and Asp solution to develop DPP-Asp-hEGF for regeneration of epithelium in conjunctival defects. The efficiency of decellularization and crosslinking and biocompatibility were evaluated in vitro, and in vivo tests were performed using a bulbar conjunctival defect rabbit model to evaluate scaffolds as a potential substitute for conjunctival reconstruction in vivo.

## 2. Materials and Methods

### 2.1. Ethics

Human amniotic membrane was collected from human placentas after cesarean deliveries under sterile conditions, which obtain approval from the Ethics Committee of the First affiliated Hospital of Jinan University, China (Ethical Code: IR. KY-2021-032). All placenta donors were serologically negative for human immunodeficiency virus, hepatitis virus types A and B, HIV, and syphilis. AM was washed with phosphate-buffered saline (PBS) containing 10% penicillin/streptomycin (P/S; Merck, Darmstadt, Germany) until cleared and shaped into a 9.0 mm diameter circle using a trephine. AM was stored in Dulbecco's modified Eagle's medium (Merck, Darmstadt, Germany) mixed 1 : 1 with glycerol (Sigma-Aldrich, St. Louis, MO, USA) for storage at −20°C and washed with PBS before use.

All experiments were approved by the Ethics Committee of Jinan University, China (Ethical Code: 20200323-21).

### 2.2. Tissue Decellularization

Native porcine pericardium (NPP) was harvested from a local slaughterhouse and was stored at −80°C for long-term storage or 4°C for short-term storage. Remove adipose tissue before decellularization; NPP were washed in PBS containing 5% P/S for 2 h, after which were soaked in bicarbonate-mixed salt solution containing 200 U/ml PLA2 (Sigma-Aldrich) and 0.5% (*w*/*v*) sodium deoxycholate (SD; Sigma-Aldrich) under continuous shaking for 2 h at RT. Then, it was followed by rinsing with bicarbonate-mixed salt solution 10 times at RT in a constant-temperature shaking water bath, 2 h each. The tissue was transferred into the 200 units/m deoxyribonuclease solution, incubated at 37°C for 6 h. Finally, the samples were washed with PBS 20 times under shaking conditions, 30 min each.

All DPP were shaped into a 9.0 mm diameter circle before being air-dried naturally and sterilized by *γ* irradiation with 25 kGy (Huada Biotechnology Co., Ltd. Huangpu, Guangzhou) and placed onto nylon carrier papers, before use.

#### 2.2.1. Histological Assessment

The samples (NPP and DPP) were fixed in 10% (*v*/*v*) neutral buffered formalin (Solarbio, Beijing, China) overnight at RT, dehydrated, and embedded in paraffin wax. All the paraffin-embedded sections were cut into 5 *μ*m sections. Slides were dewaxed and then stained for hematoxylin and eosin (Servicebio) and Masson staining (Servicebio) according to routine procedures.

#### 2.2.2. Component Analysis

For immunofluorescence, the tissue sections underwent antigen retrieval with sodium citrate buffer at 90°C for 20 min. Primary antibodies were diluted in blocking solution, and sections were incubated with primary antibodies for collagens I and IV, laminin, and fibronectin diluted at 1 : 200 (Servicebio) overnight at 4°C, followed by incubation with the secondary antibody diluted at 1 : 1000 (Servicebio), at 37°C for 2 h, followed by incubating with DAPI solution diluted at 1 : 200 (Servicebio) at RT for 10 min, kept in a dark place. The sections were observed under a fluorescence microscope, and images were collected (Leica Microsystems, Wetzlar, Germany).

The dry weight of NPP and FPP was recorded. The DNA extraction protocol followed the manufacturer instructions of the DNA extraction kit (TIANGEN, Beijing, China), and the DNA concentration was determined with a NanoDrop spectrophotometer (Thermo Scientific, Massachusetts, USA). Tissue DNA content was calculated according to the DNA concentration and sample weight (mg DNA/g tissue).

Type I collagen content (10 samples per group) was measured spectrophotometrically using a collagen quantitation kit (Jiancheng, Nanjing, China) according to the manufacturer's protocol, and the absorbance of the samples was then measured at 450 nm. Type I collagen content was expressed as ng/mg.

The number of glycosaminoglycans (GAGs) in the tissue samples of normal (*n* = 6) and decellularized (*n* = 6) was measured using the Glycosaminoglycan Kit (Biocolor, Antrim, UK). GAG content was expressed as ng/mg.

#### 2.2.3. Ultrastructural Analysis

For scanning electron microscopy (SEM), samples (NPP and DPP) were fixed by electron microscopy fixative (Servicebio) for 2 hours at RT and then postfixed with 1% OsO4 (Ted Pella Inc., USA) for 1 h at RT after washing in PBS (pH 7.4) for 3 times, 15 min each. Samples were subsequently dehydrated in increasing concentrations of ethanol: 50%, 70%, 90%, and 95% for 15 min each and, finally, in 100% ethanol with 2 changes of 15 min each. Following this, the samples were dry with a critical point dryer and attached to metallic stubs using carbon stickers and sputter-coated with gold for the 30 s. A scanning electron microscope (Hitachi, Tokyo, Japan) was used to take images.

For transmission electron microscopy (TEM), the samples were cut into a small size of 1 mm^3^ in the fixative and fixed in an EP tube with fresh TEM fixative (Servicebio). Fix in 1% OsO4 (Ted Pella Inc., USA) for 2 h at RT, and then, remove OsO4 with PBS (pH 7.4) for 3 times, 15 min each. Dehydrate in graded ethanol: 30%, 50%, 70%, and 80% for 20 min, respectively, and in 100% ethanol with 2 changes of 20 min each; and finally, the samples were put in acetone with 2 changes of 15 min each. Followed by embedding with resin and polymerization, polymerized blocks were sectioned at 60-80 nm on an Ultramicrotome (Leica) and then placed on 150-mesh cuprum grids with formvar film, stained with 2% uranium acetate saturated alcohol solution for 8 min in the dark, followed by 6% lead citrate. The cuprum grids are observed under TEM and images were taken (Hitachi).

#### 2.2.4. Cytotoxicity Assay

To determine the potential cytotoxicity of the decellularized tissue, rabbit conjunctival epithelial cells (RCEs) were incubated with DPP immersion and culture medium. For the generation of DPP extracts, pieces with a diameter of 9 mm were incubated in 1.5 ml KCM medium at 4°C for 48 h.

RCEs were isolated from rabbit conjunctival and then cultured using the tissue explant adherent method. Briefly, rabbit bulbar conjunctiva with a size of about 2∗2 cm diameter was attached into well of 6-well plates for 2 h, at 37°C; after that, it was seeded with culture medium supplemented with 4.5 g/l high-glucose Dulbecco's modified Eagle's medium (Gibco, Life Technologies) mixed 10% (*v*/*v*) fetal bovine serum (FBS; Sigma-Aldrich), 1% antibiotic–antimycotic solution (Gibco), 1% (*v*/*v*) insulin (Merck Millipore, Massachusetts, USA), 1% (*v*/*v*) NEAA (Corning, Manassas, USA), 1% (*v*/*v*) L-glutamine (Corning), 0.1% (*v*/*v*) hydrocortisone (Macklin, Shanghai, China), 0.1% (*v*/*v*) transferrin (Sigma-Aldrich), and 0.02% (*v*/*v*) epidermal growth factor (PeproTech, Rocky Hill, USA). RCEs were passaged using 0.25% Trypsin-EDTA (Gibco) and seeded in a 96-well plate at a density of 1.0–1.5 × 10^3^ cells/well in culture medium. Five replicates were set for each group.

The activity of the cells was quantitatively determined at 7 consecutive days by CCK-8 kit (Dojindo Molecular Technology, Kumamoto, Japan). The optical density (OD) value of absorbance at 450 nm was measured by a microplate reader (BioTek, Synergy H1, USA). All constructions were cultured at 37°C in a humidified atmosphere containing 5% CO_2_.

### 2.3. Crosslinking Process

The DPP was incubated in EDC/NHS (Thermo Scientific, Massachusetts, USA) solution for 20 min after full rehydration, followed with immersion in a 60 mg/ml saturated Asp solution by ultrasound with 37 Hz for 4 h and then sequentially incubated for 20 h under shaking at RT. Then, the DPP-Asp was immersed in 200 ng/ml hEGF (Thermo Scientific) solution at 4°C overnight, and DPP-Asp-hEGF was prepared. These scaffolds are stored at −20°C until use.

#### 2.3.1. Fourier Transform Infrared Spectroscopy and X-Ray Diffraction

Fourier transform infrared (FTIR) spectroscopy was performed on an FTIR spectrometer (Bruker, Karlsruhe, Germany) over the wavenumber range of 4000 to 500 cm^−1^ with a resolution of 4 cm^−1^ and scan number 64.

To further analyze structural characterization before (as reference) and after crosslinking, X-ray diffraction (Bruker) was performed in the range of 2*θ* = 0–60°.

### 2.4. Evaluation of DPP-Asp-hEGF In Vivo

#### 2.4.1. Surgical Procedure

DPP-Asp-hEGF showed a better biologically active property and was used for all further in vivo experiments consequently.

New Zealand white rabbits with a weight of 2.5 kg were chosen, and all experiments were conducted in accordance with the ARVO statement for the use of animals in ophthalmic research. Rabbits were anesthetized using 5 mg/kg zoletil (Virbac, Carlos, France), and oxybuprocaine eye drops (Santen, Osaka, Japan) were additionally applied for topical anesthesia during surgery.

A bulbar conjunctival defect of 7.5 mm diameter in the upper temporal part of the eye at a distance of 2 mm from the limbus was performed using a trephine. Rabbits were divided into three groups, with 6 rabbits per group. Defects were covered with DPP-Asp-hEGF, AM, or the sham operation (sham group). All transplants were sutured with eight single stich sutures using Vicryl 9-0.

Postoperatively, Tobramycin eye drops (Alcon, Fort Worth, USA), Tobramycin ointment (Alcon), and 0.5% Levofloxacin eye drops (Santen) were given one time every day for one week.

#### 2.4.2. Clinical Follow-Up

Observation time points of postoperative days 3 d, 7 d, 14 d, 21 d, and 28 d were chosen for all following clinical experiments.

The clinical signs of graft lysis and tearing of sutures were observed and counted. Conjunctival hyperemia was graded using the MacDonald–Shadduck scoring system and was documented by the same experimenter: grade 0, no hyperemia; grade 1, slight hyperemia; grade 2, moderate hyperemia; and grade 3, severe hyperemia. Lissamine staining (Thermo Scientific) was performed, and images were taken for the evaluation of the epithelialization using ImageJ. The defect size immediately after surgery was set as 100%.

#### 2.4.3. Histological Evaluation

The tissues were collected 28 days after surgery and fixed in 10% (*v*/*v*) neutral buffered formalin (Solarbio), and then, paraffin-embedded tissues were deparaffinized, rehydrated, and subsequently stained using HE staining, Masson staining, and PAS staining following standard protocols. Images were captured under a light microscope (Olympus, Tokyo, Japan).

#### 2.4.4. Goblet Cell Count

The number of goblet cells was assessed on PAS-stained tissue sections. The microscopic images were selected using the CaseViewer program (CaseViewer 2.3; 3DHISTECH, Budapest, Hungary) at ×200 magnification. Five different sections of each group were selected randomly and the averages calculated manually (cells/per vision).

#### 2.4.5. Epithelium Thickness

Tissue sections stained with HE were used to evaluate the thickness of the conjunctival epithelium. In each sample (5 from each group), images were selected at ×40 magnification, and 3 measurement points per transplant were carried out randomly using the CaseViewer program (CaseViewer 2.3; 3DHISTECH, Budapest, Hungary). Epithelium thickness was expressed as *μ*m.

### 2.5. Statistical Analysis

The data are expressed as the means ± standard deviation. IBM SPSS 22 (IBM, New York, USA) and GraphPad Prism 8 (La Jolla, CA, USA) were used for all statistical analyses, and one-way ANOVA or independent *T*-test was performed to compare mean values between the groups. A value of *p* < 0.05 was considered to indicate a significant difference. *p* values were designated as ^∗^*p* < 0.05, ^∗∗^*p* < 0.01, ^∗∗∗^*p* < 0.001, and ^∗∗∗∗^*p* < 0.0001.

## 3. Results

### 3.1. Evaluation of DPP In Vitro

#### 3.1.1. Characterization of DPP

**Figure 1:**
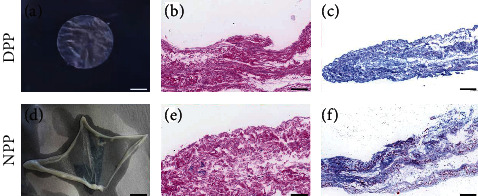
Histological analysis of (a–c) DPP and (d–f) NPP. (a, d) Macromorphology showed no significant difference between DPP and NPP, but whitened in DPP. (b, e) HE staining demonstrated no remaining nuclear components in the porcine pericardium after decellularization. (c, f) Masson staining displayed no clear difference in the morphology of collagen fibrils, organization into fibers, or the overall architecture of the collagen matrix evident after decellularization; *n* = 3. Scale bar (a–f): 50 *μ*m.

Following decellularization and air drying, the porcine pericardium showed no significant difference in terms of macromorphology, but whitened (Figures 1(a) and 1(d)). Decellularization efficiency was determined by evaluating HE staining, Masson staining, and DNA content. HE staining appeared homogeneous with pink cytoplasm in NPP and DPP, but no nuclear staining in DPP (Figures 1(b) and 1(e)). The results of Masson staining were in accordance with those of HE; as the fibrous tissue stained a light blue, no clear difference in the morphology of collagen fibrils, organization into fibers, or the overall architecture of the collagen matrix was evident after decellularization (Figures 1(c) and 1(f)).

**Figure 2:**
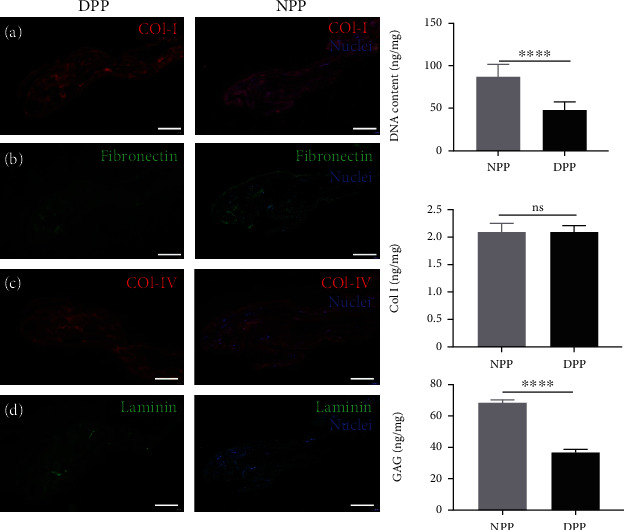
Component comparison of DPP and NPP. (a) Fluorescence staining of (A) type I collagen, (B) fibronectin, (C) collagen IV, and (D) laminin in DPP and NPP. Nuclei were stained by DAPI (scale bar = 50 *μ*m). Comparison of DPP and NPP regarding (b) DNA content, (c) type I collagen content, and (d) GAG content (^∗∗∗∗^*p* < 0.0001, ns = nonsignificant).

Immunofluorescence results showed that NPP and DPP both positively expressed type I collagen, type IV collagen, laminin, and fibronectin (Figure 2(a)).

DNA quantification study was performed on the ECM before and after decellularization. There was a decrease in DNA content after decellularization, from 86.50 ± 14.70 ng/mg in the native control to 48.24 ± 8.59 ng/mg in the DPPs (Figure 2(b)).

Quantitative analysis showed the average type I collagen of DPP was 2.08 ± 0.11 ng/mg, with no significant difference from the NPP (2.09 ± 0.15 ng/mg, *p* > 0.05, Figure 2(b)). But the GAC content was reduced by about 47% (DPP 36.33 ± 2.88 ng/mg; NPP 68.54 ± 1.74 ng/mg; ^∗∗∗∗^*p* < 0.0001).

#### 3.1.2. Microstructure of DPP

**Figure 3:**
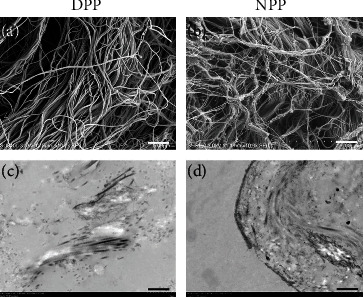
Ultrastructural analysis of (a, c) DPP and (b, d) NPP. (a, b) Scanning electron microscopy images and (c, d) transmission electron microscopy images showed the intact collagen fibrils after decellularization (scale bar (a, b) = 5 *μ*m, scale bar (c, d) = 1.0 *μ*m).

The microstructures of the scaffolds were characterized by SEM and TEM, as shown in Figure 3. The SEM images revealed that the histoarchitecture of the DPP was maintained the same as the NPP and the cell structure was not detected (Figures 3(a) and 3(c)).

Ultrastructure analysis by TEM further revealed that stromal cells were embedded in the extracellular matrix (ECM) fibers before decellularization. Following decellularization, the ultrastructure and arrangement of the collagen fibers in the DPP were similar to those in the NPP (Figures 3(b) and 3(d)), which indicates that our decellularization protocol maintained the structural extracellular matrix components intact and integrity is revealed after decellularization.

#### 3.1.3. Cytocompatibility and Cytotoxicity of the DPP In Vitro

**Figure 4:**
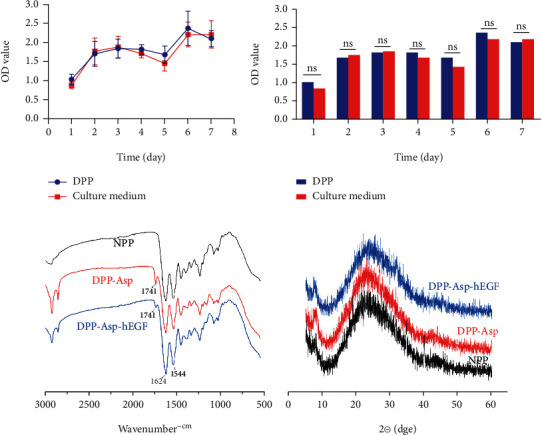
In vitro cytocompatibility of the DPP and effect of crosslinking. (a, b) CCK-8 analysis of cells on DPP extract and culture medium for 7 consecutive days after seeding. (c) Peaks of FTIR spectra of DPP, crosslinked Asp, and crosslinked Asp-hEGF. (d) XRD patterns were collected from the above samples.

CCK-8 assays were conducted to determine the viability of RCEs in DPP. Results of the CCK-8 assay showed that the OD (optical density) values increased with incubation time (Figure 4(a)) and no difference of proliferation vitality between DPP and petri dishes (Figure 4(b)) (ns = nonsignificant).

#### 3.1.4. Efficacy of Crosslinking

Samples, including DPP, DPP-Asp, and DPP-Asp-hEGF, were analyzed by FTIR spectroscopy to obtain evidence of functional groups present in the materials. FTIR investigation showed that samples (DPP-Asp and DPP-Asp-hEGF) had chemical bond vibration peaks at 1544, 1624, and 1741 cm^−1^ (Figure 4(c)), indicating the presence of amide bonding with C=O stretching and N–H bending within the amide bond.

The XRD results indicated that the Asp and hEGF were successfully loaded onto the DPP carrier. The results were further confirmed by XRD (Figure 4(d)) in which amorphous scattering peaks were detected.

### 3.2. Evaluation of DPP-Asp-hEGF In Vivo

**Figure 5:**
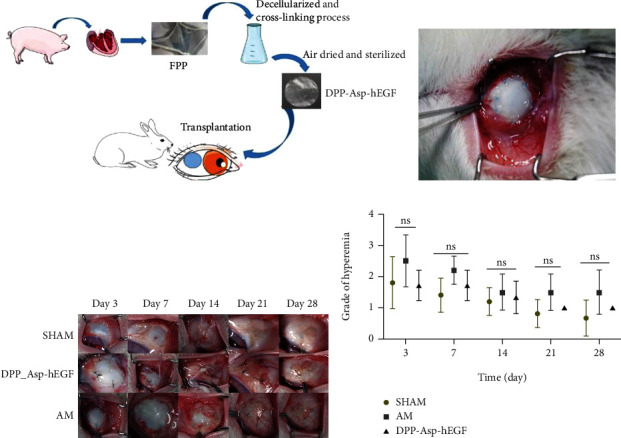
Clinical findings in rabbit conjunctival defect model. The surgical procedure (a). Scaffold transplantation on the day of surgery (b). Gross views of clinical signs at different points in time (c). A line graph showing the variation in the grade of hyperemia in each group at the different time points (d). ns = nonsignificant.

To determine the potential of the scaffold as a therapeutic material for the treatment of conjunctival reconstruction, a conjunctiva defect model was studied with New Zealand white rabbits. The surgical procedure for conjunctival reconstruction is summarized in Figure 5(a).

#### 3.2.1. Clinical Follow-Up

Similar conjunctival mild edema and hyperemia induced by the surgery were observed in all groups at the early postoperative period (one week), gradually decreasing with prolonged time in Figures 5(c) and 5(d). The reduction of the lissamine-stained wound area was revealed in all groups at day 3 postsurgery.

During the entire observational period, all the DPP-Asp-hEGF grafts were stable during wound healing, and a small amount of newly formed neovascularization was seen around the defect margin on the 14th postoperative day. However, we found that the degradation of AM began earlier than did that of DPP-Asp-hEGF. Degradation of the transplanted AM and scar formation began on day 14 in two of the six rabbits and continued until day 21. Meanwhile, the white secretion in AM groups can be seen after day 14 postoperatively. Contraction of the wound edges as shown in the ungraft groups indicated the growth of fibrous scarring.

#### 3.2.2. Epithelialization

**Figure 6:**
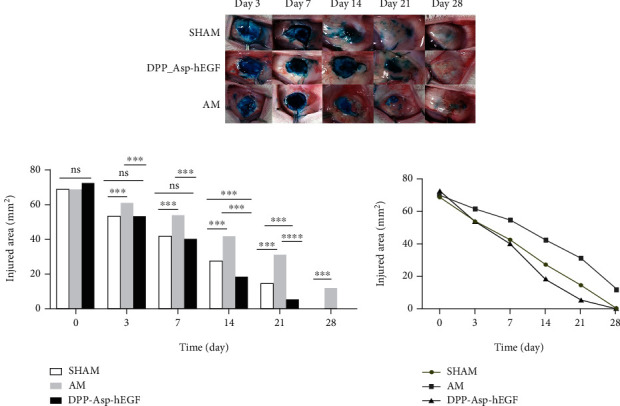
Reepithelialization in rabbit conjunctival defect model. The epithelial defects were stained with lissamine staining in each group at the various time points (a). (b) Histogram and (c) line graph showing residual epithelial defect area during process of repair, respectively (^∗∗∗^*p* < 0.005, ^∗∗∗∗^*p* < 0.001; ns = nonsignificant).

The size of the conjunctival defect area was eventually 70.63 ± 3.10 mm^2^ because of contracture formation, which is essentially equal to the size of grafts. There was no statistically significant difference in the defect area between the three groups preoperatively (*p* > 0.05) (Figure 6(b)). Three days postoperatively, neither DPP-Asp-hEGF nor AM or sham group showed a significant reduction of the lissamine-stained area of the defect compared to the initial measurement (DPP-Asp-hEGF: 53.47 ± 1.52 mm^2^, *p* < 0.001; sham: 53.68 ± 1.70 mm^2^, *p* < 0.001; 61.40 ± 3.48 mm^2^, *p* < 0.001), while the defect size between DPP-Asp-hEGF and AM was not significantly reduced (*p* > 0.05). On subsequent days, the epithelial defect reduced, especially during 1-2 weeks, and defects were completely in all rabbit eyes by day 28 in the DPP-Asp-hEGF groups and the sham groups. However, the AM groups have residual lissamine-stained area of the defect (11.90 ± 3.33 mm^2^) at day 28 (Figures 6(b) and 6(c)).

#### 3.2.3. Histological Evaluation

**Figure 7:**
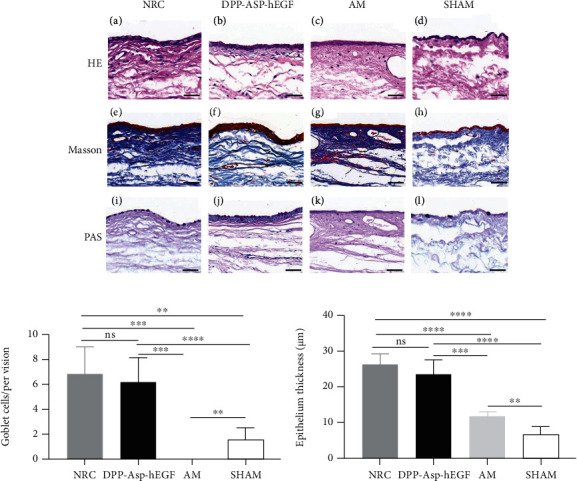
Histological analysis of scaffolds transplanted in a rabbit conjunctival defect model in 28 days postoperatively. (a) The (A–D) HE staining, (E–H) Masson staining, and (I–L) PAS staining of (A, E, I) NRC or (B, F, J) DPP-Asp-hEGF or (C, G, K) AM or (D, H, L) sham conjunctival defects. Quantitative results of (b) repaired goblet cells and (c) epithelium thickness among three groups (data represented as mean value ± SD; ^∗∗∗^*p* < 0.005, ^∗∗∗∗^*p* < 0.001; scale bar = 50 *μ*m).

HE staining of the DPP-Asp-hEGF revealed 3–5 cell layer thickness (223.68 ± 3.97 *μ*m), consistent with normal conjunctiva tissue (26.61 ± 2.86 *μ*m) (Figures 7(a) and 7(c)). With the observation, it was shown that significantly fewer epithelial cells were found in the AM (11.85 ± 1.31 *μ*m) and sham (7.00 ± 1.92 *μ*m) groups, though with complete wound healing in the sham groups according to lissamine staining.

Masson staining exhibited a loose network of collagen fibers and vessel filled red blood cells in the DPP-Asp-hEGF (Figure 7(a)). In contrast, neovascularization is barely detectable in sham, and dense collagen fibrils, which are associated with fibrotic scarring, were presented in AM (Figure 7(a)).

The number of PAS-stained goblet cells in the DPP-Asp-hEGF group was significantly higher than that in the AM group and the control, indicating DPP-Asp-hEGF scaffold could effectively achieve functional recovery of the conjunctiva. Quantitatively, there were no significant changes in goblet cell amount of the DPP-Asp-hEGF group and the NRC group, but no goblet cells were found in the AM (NRC: 6.80 ± 2.17; DPP-Asp-hEGF: 6.20 ± 1.92; AM: 0.00 ± 0.00; sham: 1.60 ± 0.89, goblet cells/per vision) (Figure 7(b)).

## 4. Discussion

In this study, we aim to develop a promising scaffold for conjunctiva reconstruction to recover mucin secretion from conjunctival goblet cells. Extracellular matrix (ECM) mainly contains proteoglycans such as GAGs (glycosaminoglycans) and proteins such as collagen and elastin produced by fibroblasts [23]. On the other hand, decellularized matrices that contain low doses of native growth factors are clinically used as a graft substitute for chronic wounds. The porcine ECM was found to have a high degree of sequence and domain structural homology with humans and could potentially be utilized as scaffold materials in tissue engineering [13], such as heart valves and dermis [24]. The pericardium contains extracellular connective components, including elastin, collagen, and proteoglycans, and provided better biocompatibility and mechanical strength than a pure collagen film [25]. Acellular bovine pericardium grafts have been used in ocular surface reconstruction as a substrate for conjunctiva with encouraging results [15], but there is a lack of research about decellularized porcine pericardium use in conjunctival reconstruction.

Since the first introduced amnion membrane to conjunctival reconstructive surgery in 1940 by De Roth [26], the amniotic membrane had been employed in treatments of different ocular diseases and the reconstruction of damaged tissue for many decades. However, for severe ocular surface disease without cells of surrounding healthy conjunctiva retained, simple AM transplantation is challenging [27]. There is an increasing amount of evidence pointing to accelerated degradation of AM in an inflamed surrounding that results in incomplete epithelialization and inadequate numbers of goblet cells [13, 28]. Some cytokines and growth factors, such as interleukin-1 or TGF-*β*, induced by AM may be a potential risk factor for postoperative symblepharon and scar formation [12].

Therefore, searching for alternative sources for conjunctival reconstruction to overcome the above limitations of AM therapy is urgently needed. Although a few preliminary studies had reported the use of artificial synthetic materials, as a substitute for conjunctival reconstruction and played a positive role in short-term effects, synthetic polymer materials also have disadvantages in terms of poor biocompatibility, inferior tensile strength, and lack of 3D microenvironment [29, 30]. Decellularized ECM is a biomaterial that best mimics the native cellular microenvironment and was regarded as a promising scaffold because of good bioactivity and biodegradability and well tolerated in terms of biocompatibility and immune reactions [31, 32].

In the past times, the use of decellularized ECM in the setting of conjunctival reconstruction has shown promising data in the ocular reconstruction, but most of them did not meet the conjunctival goblet cell criteria required for conjunctival reconstruction [12, 13, 15]. Functional restoration of goblet cells may be a critical procedure for the reconstruction of the ocular surface [33]. Thus, to maintain the normal structure and function of ocular surface epithelium, we established chemical crosslinking for the optimization of the decellularized process, loading hEGF on scaffolds via chemical bonds of Asp. Further studies with animal experiments were implemented to evaluate the performance of DPP-Asp-hEGF.

In our study, the porcine pericardium was selected as scaffold material and our decellularization protocol effectively removed porcine cellular material, supported by 48.24 ± 8.59 ng/mg in DNA content observed. Results from in vivo studies suggested that decellularized tissue containing less than 50 ng/mg of DNA is adequate [34, 35]. Currently, there is no universally accepted, standardized protocol for decellularization [35]. It appears that combined methods could be more useful to perform efficient decellularization than treatment alone [36]. Phospholipase A2 (PLA2) is a kind of hydrolase that acts on ester bonds and completely hydrolyzes triglycerides into fatty acids and glycerol. PLA2 has been proven to be an efficient decellularized method for the preparation of scaffolds [21, 37] and has the advantage of maintaining ECM and collagenous structure [36]. Moreover, the absence of nuclear structures in HE staining was an indicator of effective cell removal during the decellularization process, and the extracellular matrix components type I collagen, type IV collagen, and laminin do not appear to be disrupted following decellularization according to qualitative and quantitative data (Figure 2). These contents were ideally suited as 3D cell culture model systems mimicking the ECM and could be further supplemented with ECM proteins or adhesive peptides [38]. As shown in Figure 3, results revealed by electron microscopy that the structure of the DPP was intact, and the epithelial cells were not identified. Meanwhile, low-molecular-weight PLA2 was thought to have no biological toxicity on cells because it is easier to be washed and reduced the residual on scaffolds [36]. CCK-8 assay results showed no difference between DPP scaffolds and controls (*p* > 0.05) (Figures 4(a) and 4(b)).

To improve hydrophilicity and biocompatibility of scaffold and maintain the original nanofiber structure, we crosslinked EDC/NHS with aspartic acid (Asp) to form amide groups (amide A) in the DPP samples. For DPP-Asp-hEGF, a new peak was observed in the region 1544–1741 cm^−1^, which is associated with a primary amine. Meanwhile, with utilization of carboxyl groups from Asp, hEGF were successfully crosslinked to DPP-Asp (Figure 4(c)). Prior reports showed that crosslinked tissues had biological properties closer to those of native tissues [16], and therefore, the cell sheet cultured on these tissues was considered ideal. Meanwhile, chemical crosslinking will significantly inhibit the degradation of biological scaffolds and alter the host tissue response to the biological material [16]. Asp is a nontoxic, biocompatible, and biodegradable protein widely expressed in the human body and may help to promote damage repair and the production of extracellular matrix [21]. In this study, Asp was crosslinked with collagen to form hydrogen bonds and was used as a spacer arm to crosslink hEGF on DPP-Asp. It was shown that hEGF acts on receptor and activates its kinase activity and then initiates DNA synthesis to induce the repair of defective tissues. Thus, hEGF has a very good activity to promote cell proliferation and promote the regeneration of epidermal cells during wound healing [39].

The stability and biocompatibility of conjunctival equivalents should be proven in vivo (Figure 5). All groups showed the inflammatory response with different degrees during the follow-up, and there were no statistically significant differences in hyperemia index among the groups (Figure 5(c)). DPP-Asp-hEGF showed reduced suture loss and superior integration during follow-up, whereas more inflammatory cells were found histologically in the DPP-Asp-hEGF groups on postoperative day 28 (Figure 6(a)); this might be induced by the material degradation.

Furthermore, the DPP-Asp-hEGF groups represented faster repair rates of defect injury than a sham, the slowest rate observed in AM (Figures 6(b) and 6(c)).

In addition to reepithelialization, conjunctival wound healing involves fibroblast proliferation and extracellular matrix deposition [40]. Fibronectin induced by myofibroblasts displayed traction forces for wound closure [41], and ECM scaffolds lead to the deposition of host-derived neo matrix and eventually constructive tissue remodeling with a minimum of scar tissue [42]. During wound healing, myofibroblasts tend to migrate into well-integrated grafts, which indicated a well-integrated transplant might be superior in healing wounds [4]. From Masson staining (Figure 7(a)), loose, randomly aligned collagen fibers were revealed in the DPP-Asp-hEGF after 28 days, which were characteristic of native conjunctiva and also indicate transplantations were well attached to the defective sites. Furthermore, we thought that the degradation rate of DPP-Asp-hEGF was sufficient to stably hold the transplantations for complete conjunctiva reconstruction after chemical crosslinking. Meanwhile, the collagen and basement membrane of DPP-Asp-hEGF would provide migration for epithelial cells in the processes of reepithelialization. However, in this study, AM treatment showed a slower rate of healing, even incomplete epithelialization at 28 days. Therefore, we surmised that the absence of neovascularization and dense collagen fibrils, which might slow down the nutrition transport needed for reepithelialization, may explain the inconsistent clinical outcomes after AM transplantation.

Conjunctival goblet cells secrete mucins that are glycoproteins that play an important role in the ocular surface barrier function [43]. Numerous studies have revealed that ECM from decellularized tissues may provide a vital and functional microenvironment to support the growth and differentiation of goblet cells [44]. Comparable goblet cells embedded in the epithelium were observed on top of DPP-Asp-hEGF, revealing no significant difference in the average count compared with NRC at day 28 (NRC: 6.80 ± 2.17, DPP-Asp-hEGF: 6.20 ± 1.92, counts/per vision, *p* > 0.05). But there were no goblet cells and multilayered epithelium was observed on AM in our present study, which may be the result of poor integration or donor variation [45].

In this work, DPP-Asp-hEGF showed superior efficiency in terms of the reepithelialization and inhibition of scar formation. There are still some shortcomings that need further investigation, such as the effects of scaffolds on the secretory function of conjunctival goblet cells, clinical application value for extensive conjunctival damage, and severe inflammation conditions.

## 5. Conclusions

In our study, the decellularization of porcine pericardium displayed favorable biological and biochemical properties in vivo. In addition, the biocompatible DPP-Asp-hEGF achieved better formation of multilayered epithelium including goblet cells and superior integration with native tissue during follow-up within 28 days in vitro. Thus, DPP-Asp-hEGF can be a better appropriate option for the successful reconstruction of the conjunctiva.

## Data Availability

The data used to support the findings of this study are included in the article.
